# Lidocaine Attenuates miRNA Dysregulation and Kinase Signaling Activation in a Porcine Model of Lung Ischemia/Reperfusion Injury

**DOI:** 10.3390/ijms262110385

**Published:** 2025-10-25

**Authors:** Alberto Alonso, Sergio D. Paredes, Agustín Turrero, Lisa Rancan, Ignacio Garutti, Carlos Simón, Elena Vara

**Affiliations:** 1 Department of Cardiovascular Surgery, 12 de Octubre University General Hospital, Avda. de Córdoba, s/n, 28041 Madrid, Spain; aalonso@salud.madrid.org; 2 Department of Physiology, School of Medicine, Complutense University of Madrid, Avda. Complutense, s/n, 28040 Madrid, Spain; 3 Departmental Unit of Biostatistics—Department of Statistics and Operations Research, School of Medicine, Complutense University of Madrid, Avda. Complutense, s/n, 28040 Madrid, Spain; turrero@ucm.es; 4 Department of Biochemistry and Molecular Biology, School of Medicine, Complutense University of Madrid, Avda. Complutense, s/n, 28040 Madrid, Spain; lisaranc@ucm.es (L.R.); evaraami@ucm.es (E.V.); 5 Department of Anesthesiology, Gregorio Marañón University General Hospital, C/Doctor Esquerdo, 46, 28007 Madrid, Spain; igarutti@ucm.es; 6 Department of Pharmacology and Toxicology, School of Medicine, Complutense University of Madrid, Avda. Complutense, s/n, 28040 Madrid, Spain; 7 Department of Thoracic Surgery, Gregorio Marañón University General Hospital, C/Doctor Esquerdo, 46, 28007 Madrid, Spain; cmsimon@ucm.es; 8 Department of Surgery, School of Medicine, Complutense University of Madrid, Avda. Complutense, s/n, 28040 Madrid, Spain

**Keywords:** ischemia/reperfusion injury, lung transplantation, lidocaine, microRNAs, MAPK signaling, p-38 MAPK, ERK, PI3K, AKT, porcine model

## Abstract

Ischemia/reperfusion (I/R) injury is a major complication in lung transplantation. Recent evidence suggests that mitogen-activated protein kinases (MAPKs) such as p-38 mitogen-activated protein kinase (p-38 MAPK) and extracellular signal-regulated kinase (ERK), along with functionally related kinases like phosphoinositide 3-kinase (PI3K) and protein kinase B (AKT), contribute to I/R pathophysiology by mediating inflammatory and stress-response signaling. MicroRNAs (miRNAs) also play a regulatory role in these processes. Lidocaine has demonstrated anti-inflammatory activity in several tissues; however, its ability to modulate miRNA expression and kinase activation in the lung is not yet fully understood. This study investigated the involvement of these signaling molecules in lung I/R injury and evaluated the modulatory effect of intravenous lidocaine in a porcine lung auto-transplantation model. Eighteen large white pigs were assigned to sham-operated (*n* = 6), control (lung auto-transplantation, *n* = 6), or lidocaine-treated (*n* = 6) groups. Lidocaine was administered as a 1.5 mg/kg bolus followed by a continuous infusion (1.5 mg·kg^−1^·h^−1^). Lung biopsies were collected before ischemia, before reperfusion, and at 30- and 60-min post-reperfusion to assess total and phosphorylated levels of p-38 MAPK, ERK, PI3K, and AKT (Thr308, Ser473), along with miR-126, miR-142-5p, miR-152, and miR-155 expression. I/R increased p-38 MAPK and AKT, and enhanced phosphorylation of all four kinases. miRNA levels were also upregulated. Lidocaine partially or completely attenuated these changes. These findings support a role for these molecular pathways in lung I/R injury and suggest that lidocaine may offer protective effects through their modulation.

## 1. Introduction

Ischemia/reperfusion (I/R) lung injury is a major contributor to primary graft failure. Despite advances in lung preservation, surgical techniques, and perioperative care, I/R injury remains one of the leading causes of early morbidity and mortality following lung transplantation [1].

MicroRNAs (miRNAs) are a large family of short (~22 nucleotides), noncoding RNAs that regulate gene expression post-transcriptionally and translationally in protozoa, plants, and metazoans [2,3,4]. They act primarily by binding with perfect or near-perfect complementarity to the 3′ untranslated regions (UTRs) of target mRNAs, resulting in mRNA degradation or translational repression [5]. Recent studies have highlighted the central role of miRNAs in modulating intracellular signaling pathways, including those involved in inflammation and stress responses during I/R injury [6]. Among them, miR-155 is one of the most extensively studied and has been shown to modulate innate immunity by promoting or stabilizing pro-inflammatory cytokine expression during inflammatory stimuli [7]. miR-152 has been implicated in the regulation of apoptosis and immune signaling [8], while miR-142-5p has been associated with hematopoietic cell function and inflammatory modulation [9]. Additionally, miR-126 plays a critical role in maintaining endothelial integrity and regulating vascular inflammation [10], mechanisms that are known to contribute to I/R-induced lung injury.

**Figure 1:**
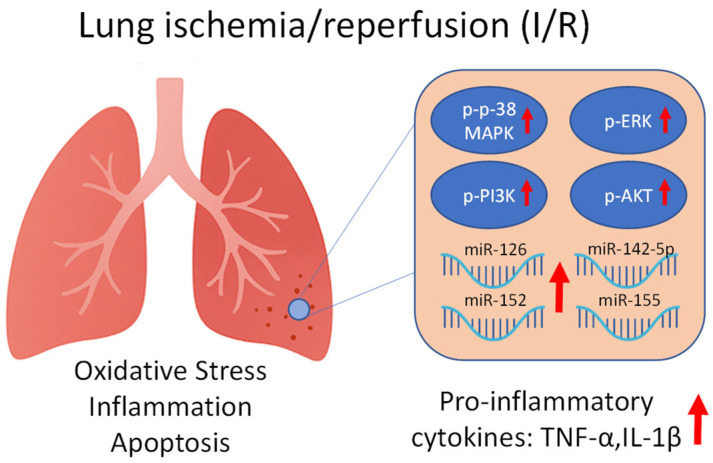
Schematic representation of the proposed interactions between oxidative stress, kinase activation, and microRNA (miRNA) expression during lung ischemia/reperfusion (I/R) injury. Oxidative stress leads to the activation (phosphorylation) of p-38 mitogen-activated protein kinase (p-38 MAPK), extracellular signal-regulated kinase (ERK), phosphoinositide 3-kinase (PI3K), and protein kinase B (AKT), which may influence the expression of specific miRNAs (miR-126, miR-142-5p, miR-152, and miR-155) involved in inflammatory and vascular responses. The red arrow indicates upregulation of the depicted elements.

Each miRNA can influence distinct cellular responses by targeting specific signaling pathways, including those governed by mitogen-activated protein kinases (MAPKs). The MAPK family includes extracellular signal-regulated kinases (ERK1/2), ERK5, c-Jun N-terminal kinases (JNK1/2/3), and p-38 isoforms (α, β, γ [also known as ERK6], and δ). Among these, p-38 MAPK has been extensively characterized for its role in inflammation. Initially identified as a kinase activated by lipopolysaccharide (LPS) and interleukin-1 (IL-1), p-38 MAPK exhibits distinct cell type-specific functions and regulates both pro- and anti-inflammatory gene expression [11]. Moreover, MAPK pathways interact with other signaling cascades such as phosphoinositide 3-kinase (PI3K) and protein kinase B (AKT), which are also involved in the regulation of inflammation, apoptosis, and cell survival during I/R injury [12] (Figure 1).

Lidocaine, a commonly used local anesthetic, has been shown to exert anti-inflammatory effects when administered intravenously [13]. These properties have led to its use in various surgical and critical care settings, where it has been associated with improved postoperative outcomes [14]. However, its potential to modulate miRNA expression and intracellular kinase activation during lung I/R injury remains poorly understood.

Based on the previously described roles of the selected miRNAs in regulating inflammatory and vascular responses, and their potential interaction with MAPK and related kinase pathways, this study investigated their involvement in lung I/R injury and evaluated whether intravenous lidocaine could modulate their expression and the activation of associated signaling proteins.

## 2. Results

No statistically significant differences were found among the experimental groups regarding animal weight or surgical duration. Likewise, pulmonary ischemia time did not differ significantly between the control and lidocaine groups. Hemodynamic variables, including mean arterial pressure (MAP), heart rate (HR), stroke volume variation (SVV), central venous pressure (CVP), cardiac index (CI), global end-diastolic volume index (GEDVI), extravascular lung water index (ELWI), and systemic vascular resistance index (SVRI), remained comparable among groups throughout the experimental protocol. Similarly, arterial blood gas parameters—partial pressure of oxygen (PaO_2_), partial pressure of carbon dioxide (PaCO_2_), arterial oxygen saturation (SaO_2_), and pH—showed no significant intergroup differences at any of the evaluated time points. Detailed results for these measurements are provided in the Supplementary Materials.

**Figure 2:**
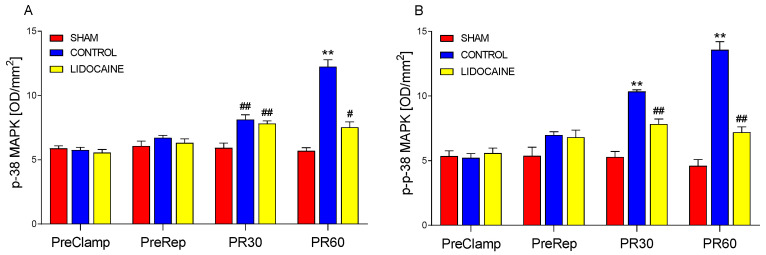
Levels of p-38 MAPK (**A**) and phosphorylated p-38 MAPK (p-p38 MAPK) (**B**) in lung biopsies collected at four time points: PreClamp (before ischemia), PreRep (before reperfusion), PR30 (30 min after reperfusion), and PR60 (60 min after reperfusion) (*n* = 6 per group). Data represent mean ± SEM. Within each time point, red bars indicate sham-operated animals, blue bars control animals, and yellow bars lidocaine-treated animals. ** *p* < 0.001 vs. both sham and lidocaine; # *p* < 0.01 or ## *p* < 0.001 vs. sham. Results are presented in optical density per mm^2^ (OD/mm^2^).

Figure 2 shows the levels of p-38 MAPK (Figure 2A) and its phosphorylated form, p-p-38 MAPK (Figure 2B), in lung biopsies collected at four experimental time points: before ischemia (PreClamp), before reperfusion (PreRep), and at 30 min (PR30) and 60 min (PR60) after reperfusion. At PreClamp and PreRep, no significant differences were observed among sham-operated, control, and lidocaine-treated groups for either form of p-38 MAPK. At PR30, p-38 MAPK levels were significantly higher in both control and lidocaine-treated animals compared to sham (*p* < 0.001), with no difference between the two experimental groups, indicating that lidocaine did not attenuate the early reperfusion-induced increase in p-38 MAPK expression. By PR60, p-38 MAPK levels followed a sham–lidocaine–control gradient, with the lowest levels in sham-operated animals, intermediate levels in the lidocaine group (*p* < 0.01), and the highest levels in the control group (*p* < 0.001), suggesting a partial inhibitory effect of lidocaine on the late-stage increase in p-38 MAPK expression. A different pattern was observed for p-p38 MAPK at PR30: phosphorylation levels were significantly elevated in both control and lidocaine-treated animals compared to sham (*p* < 0.001); however, lidocaine significantly reduced phosphorylation levels relative to control, although levels remained higher than those in the sham group. This indicates a partial attenuation of early reperfusion-induced p-p-38 MAPK activation. At PR60, the same sham–lidocaine–control gradient was observed, with all group differences reaching significance (*p* < 0.001). These findings demonstrate that lidocaine exerted a measurable inhibitory effect on reperfusion-induced activation of the p-38 MAPK pathway, particularly at the level of phosphorylation, and that this effect was more pronounced at later stages of reperfusion.

**Figure 3:**
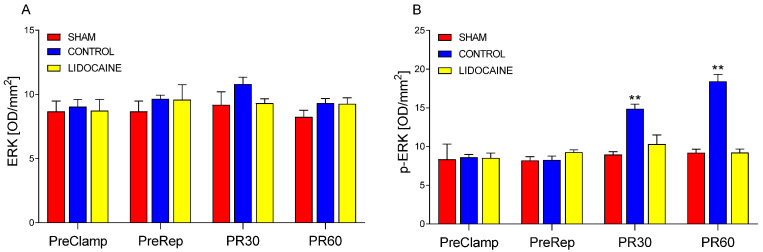
Levels of ERK (**A**) and phosphorylated ERK (p-ERK) (**B**) in lung biopsies collected at four time points: PreClamp (before ischemia), PreRep (before reperfusion), PR30 (30 min after reperfusion), and PR60 (60 min after reperfusion) (*n* = 6 per group). Data represent mean ± SEM. Within each time point, red bars indicate sham-operated animals, blue bars control animals, and yellow bars lidocaine-treated animals. ** *p* < 0.001 vs. both sham and lidocaine. Results are presented in optical density per mm^2^ (OD/mm^2^).

ERK and p-ERK levels are presented in Figure 3. ERK (Figure 3A) showed no significant differences among sham-operated, control, and lidocaine-treated animals at any of the time points. In contrast, p-ERK (Figure 3B) increased significantly in the control group at both PR30 and PR60 compared to sham (*p* < 0.001). This reperfusion-induced phosphorylation was completely blocked by lidocaine treatment, as p-ERK levels in lidocaine-treated animals remained comparable to those in the sham group. These results suggest that, although ERK remained unchanged, lidocaine fully prevented the activation of ERK signaling (p-ERK) during reperfusion.

**Figure 4:**
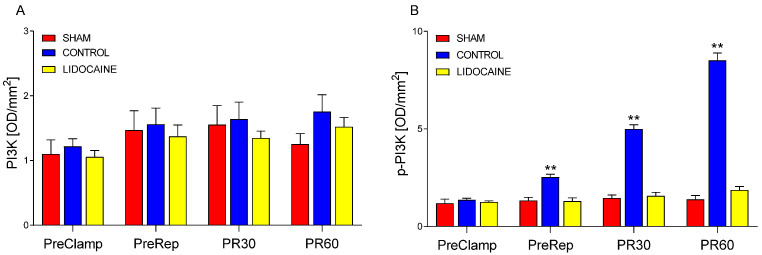
Levels of PI3K (**A**) and phosphorylated PI3K (p-PI3K) (**B**) in lung biopsies collected at four time points: PreClamp (before ischemia), PreRep (before reperfusion), PR30 (30 min after reperfusion), and PR60 (60 min after reperfusion) (*n* = 6 per group). Data represent mean ± SEM. Within each time point, red bars indicate sham-operated animals, blue bars control animals, and yellow bars lidocaine-treated animals. ** *p* < 0.001 vs. both sham and lidocaine. Results are presented in optical density per mm^2^ (OD/mm^2^).

Figure 4 displays the quantification of PI3K (Figure 4A) and its phosphorylated form (p-PI3K; Figure 4B) in lung tissue. PI3K levels remained consistent across all experimental groups and time points, indicating that its levels were not influenced by ischemia, reperfusion, or lidocaine administration. In contrast, p-PI3K increased significantly in control animals beginning at the PreRep time point and remained elevated at PR30 and PR60 compared to sham (*p* < 0.001). Lidocaine treatment effectively blocked this increase at all time points, with phosphorylation levels remaining comparable to those in the sham group. These findings indicate that lidocaine completely inhibited the activation of PI3K triggered by reperfusion, and even by ischemia, without altering its basal levels.

**Figure 5:**
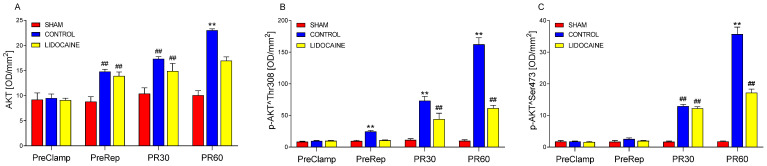
Levels of AKT (**A**), phosphorylated AKT^Thr308 (p-AKT^Thr308) (**B**), and phosphorylated AKT^Ser473 (p-AKT^Ser473) (**C**) in lung biopsies collected at four time points: PreClamp (before ischemia), PreRep (before reperfusion), PR30 (30 min after reperfusion), and PR60 (60 min after reperfusion) (*n* = 6 per group). Data represent mean ± SEM. Within each time point, red bars indicate sham-operated animals, blue bars control animals, and yellow bars lidocaine-treated animals. ** *p* < 0.001 vs. both sham and lidocaine; ## *p* < 0.001 vs. sham. Results are presented in optical density per mm^2^ (OD/mm^2^).

The levels of AKT and its phosphorylated forms were measured in lung tissue to assess activation of the PI3K/AKT signaling pathway during I/R (Figure 5). AKT is shown in Figure 5A, AKT phosphorylated at threonine 308 (p-AKT^Thr308) in Figure 5B, and AKT phosphorylated at serine 473 (p-AKT^Ser473) in Figure 5C. Phosphorylation at Thr308, primarily mediated by phosphoinositide-dependent kinase-1 (PDK1), is required for partial activation of AKT, while phosphorylation at Ser473, regulated by the mTORC2 complex, is necessary for full activation and downstream signaling. All markers were assessed at the four experimental time points, as with the markers previously presented. As shown in Figure 5A, AKT was significantly increased in the control group compared to sham at PreRep, PR30, and PR60 (*p* < 0.001), while no significant differences were observed at PreClamp among the groups. Lidocaine treatment did not affect AKT levels at PreRep or PR30, as values remained similar to those in the control group. However, at PR60, lidocaine significantly reduced AKT levels (*p* < 0.001), returning them to values comparable to sham. For p-AKT^Thr308 (Figure 5B), control animals exhibited a significant increase at PreRep, PR30, and PR60 relative to sham (*p* < 0.001), while no group differences were observed at PreClamp. Lidocaine fully blocked this increase at PreRep, with levels matching those of sham-operated animals. At PR30 and PR60, lidocaine attenuated but did not fully suppress phosphorylation at Thr308, as values were significantly lower than in the control group but remained elevated compared to sham. A distinct pattern was observed for p-AKT^Ser473 (Figure 5C): no differences among groups were found at PreClamp or PreRep. At PR30, phosphorylation levels followed the same pattern described for PR30 AKT, with increased values in control animals and no effect of lidocaine. By PR60, the pattern resembled that of P60 p-AKT^Thr308, with lidocaine partially reducing Ser473 phosphorylation, i.e., values were lower than in the control group but still higher than in sham-operated animals.

The quantitative Western blot data for p-38 MAPK, ERK, PI3K, AKT, and their phosphorylated forms across the three experimental groups are summarized in Table 1.

**Figure 6:**
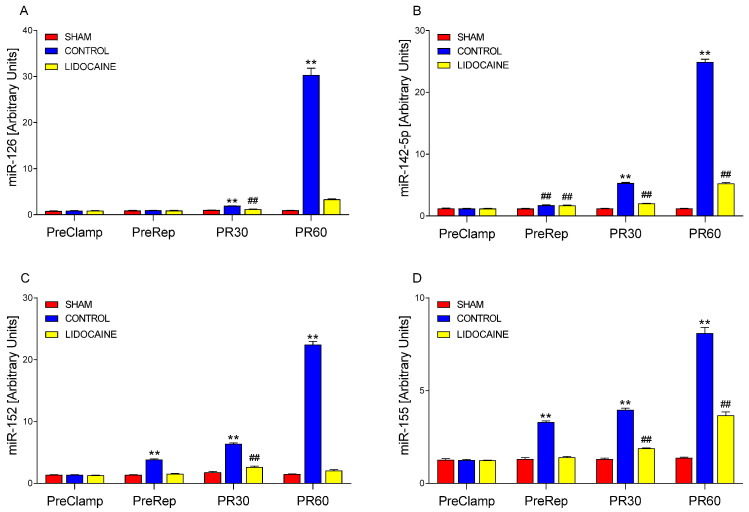
Relative expression of miR-126 (**A**), miR-142-5p (**B**), miR-152 (**C**), and miR-155 (**D**) in lung biopsies collected at four time points: PreClamp (before ischemia), PreRep (before reperfusion), PR30 (30 min after reperfusion), and PR60 (60 min after reperfusion) (*n* = 6 per group). Data represent mean ± SEM. Within each time point, red bars indicate sham-operated animals, blue bars control animals, and yellow bars lidocaine-treated animals. ** *p* < 0.001 vs. both sham and lidocaine; ## *p* < 0.001 vs. sham. Expression levels are presented in arbitrary units.

The expression patterns of miR-126, miR-142-5p, miR-152, and miR-155 across experimental groups and time points are shown in Figure 6. In Figure 6A, miR-126 expression showed no significant differences among sham-operated, control, and lidocaine-treated groups at the PreClamp and PreRep time points. At PR30, expression was significantly increased in the control group compared to both sham and lidocaine groups (*p* < 0.001). Although lidocaine group showed significantly lower expression than the control group (*p* < 0.001), levels were still elevated relative to sham (*p* < 0.001). At PR60, the control group exhibited a marked and highly significant upregulation (*p* < 0.001), while expression in the lidocaine group remained low and was not significantly different from sham. These findings indicated that lidocaine partially reduced miR-126 expression at PR30 and completely suppressed the marked increase observed at PR60.

As for the levels of miR-142-5p (Figure 6B), no differences were detected at PreClamp, but by PreRep, both control and lidocaine groups exhibited significantly higher expression than sham (*p* < 0.001). At PR30, expression increased further in the control group, remaining significantly higher than both sham and lidocaine (*p* < 0.001). The lidocaine group showed intermediate levels, significantly lower than control but still elevated compared to sham (*p* < 0.001). At PR60, the control group reached its highest expression levels (*p* < 0.001), while lidocaine significantly blunted this response yet remained higher than sham (*p* < 0.001).

Levels of miR-152 are shown in Figure 6C. At pre-reperfusion (PreRep), expression was significantly elevated in the control group compared to sham and lidocaine (*p* < 0.001), while lidocaine and sham showed no difference. During reperfusion, expression in the control groups continued to rise with respect to their sham groups (*p* < 0.001), with a moderate increase at 30 min after reperfusion and a marked peak at 60 min after reperfusion. Notably, the elevation at 60 min was much greater than at both PreRep and 30 min, indicating a strong time-dependent response, similar to the patterns observed for miR-126 and miR-142-5p (Figure 6A and Figure 6B, respectively). At PR60, lidocaine completely prevented the increase (*p* < 0.001), maintaining expression at sham levels. At PR30, lidocaine significantly reduced the response compared to control but remained higher than sham (*p* < 0.001), indicating a partial attenuation.

Figure 6D displays the expression profile of miR-155. At PreClamp, no differences were observed among sham, control, and lidocaine groups, as seen for the other three miRNAs. By PreRep, expression was significantly elevated in the control group compared to sham and lidocaine (*p* < 0.001), while the latter two did not differ. Expression in the control groups continued to rise during reperfusion, with further increases at 30 and 60 min after reperfusion (*p* < 0.001 vs. sham). Lidocaine significantly reduced miR-155 expression relative to the control group (*p* < 0.001) at both PR30 and PR60, but levels remained higher than sham (*p* < 0.001), indicating that the increase was only partially suppressed.

## 3. Discussion

### 3.1. Molecular Mechanisms Underlying I/R Injury

During lung transplantation, the damage caused by the I/R process greatly influences the outcome of the procedure. Therefore, it is a key objective to understand the underlying pathophysiological mechanisms in order to mitigate, as much as possible, the negative consequences that determine transplant success. Among the molecular mediators involved, MAPKs and miRNAs have emerged as key regulators of the inflammatory and reparative processes triggered by I/R. MAPKs regulate a multitude of signaling pathways and modulate gene expression with both pro- and anti-inflammatory outcomes [15]. These kinases are activated through phosphorylation, initiating downstream molecular cascades [16]; thus, their dysregulation may play a central role in the pathogenesis of I/R injury during lung transplantation. In parallel, miRNAs act as post-transcriptional regulators of gene expression and have been identified as important molecules involved in I/R injury in multiple organs, through their ability to alter the expression of genes associated with inflammatory pathways. Moreover, miRNAs have not only been implicated as regulators of responses to solid organ transplantation but also as potential biomarkers that may predict the patient’s response to transplantation in a simple and practical manner, as they are detectable in peripheral blood [17].

Within these signaling networks, the p-38-MAPK pathway is a well-established mediator of proinflammatory responses, and its activation has been shown to exacerbate I/R damage in the lungs [18], heart [19], and other organs. In contrast, the ERK [20] and PI3K-AKT [21] pathways have been associated with anti-inflammatory and cytoprotective effects, and may serve as therapeutic targets to promote tolerance to I/R injury.

### 3.2. Modulatory Effects of Lidocaine on MAPK and miRNA Responses

In our study, focusing on the phosphorylated forms, we found that ischemia significantly increased p-PI3K, and p-AKT^Thr308 expression in the control group. Reperfusion at 30 and 60 min further elevated these levels, including p-p-38-MAPK. In contrast, p-ERK and p-AKT^Ser473, both linked to anti-inflammatory signaling, only showed significant increases after 30 and 60 min of reperfusion, likely representing a delayed compensatory response to ongoing inflammatory damage. These findings are consistent with previous reports showing that ERK1/2 levels are elevated in p-38-MAPK-dependent I/R injury models, and that ERK activation decreases when p-38 overexpression is inhibited [18].

Efforts to attenuate I/R injury have included the use of systemic anti-inflammatory agents [22,23]. Notably, intravenous administration of lidocaine has been reported to exert anti-inflammatory effects [24]. In our model, lidocaine treatment significantly reduced p-p-38-MAPK expression at both 30 and 60 min after reperfusion (PR30 and PR60), indicating a decrease in proinflammatory and proapoptotic signaling. Although phosphorylated MAPK levels remained higher than in the sham group, they were significantly lower than in the control group, demonstrating a partial but meaningful attenuation of MAPK activation. Notably, lidocaine completely suppressed the phosphorylation of ERK and PI3K during reperfusion, an effect already evident at PR30 and even more pronounced at PR60, while AKT phosphorylation was only partially reduced. This differential effect may be due to AKT’s role as a downstream signaling hub, integrating inputs from multiple upstream pathways including PI3K, growth factors, and cytokines, which may limit the extent of its inhibition under lidocaine treatment. This interpretation is supported by studies showing that AKT is activated not only via PI3K but also through cross-talk with MAPK and mTOR pathways, and is involved in promoting cell survival, metabolic regulation, and inflammation resolution [25,26]. As such, complete inhibition of AKT is often more difficult to achieve than for upstream kinases like PI3K or ERK. Together, these observations suggest that while lidocaine strongly attenuates proinflammatory signaling, it preserves partial activity of survival pathways, potentially contributing to graft protection during reperfusion.

In parallel, to determine whether these kinase-related effects were associated with post-transcriptional regulation, we examined whether the observed changes in MAPK activation were accompanied by alterations in the expression of selected miRNAs previously shown to be modulated by I/R [27]. Among these, miR-142-5p [28] and miR-155 [29,30] are associated with proinflammatory, profibrotic, and proapoptotic activity, whereas miR-152 [31] and miR-126 are known to exert anti-inflammatory effects. Notably, miR-126 plays a critical anti-inflammatory and vasoprotective role in endothelial tissues. It maintains vascular integrity and promotes angiogenic signaling by targeting phosphoinositide-3-kinase regulatory subunit 2 (PIK3R2), a negative regulator of the PI3K/AKT pathway [32], and reduces leukocyte adhesion by suppressing vascular cell adhesion molecule-1 (VCAM-1) expression [33], thereby contributing to inflammation resolution and endothelial stability during I/R injury.

Our results were consistent with previous studies: miR-142-5p, miR-152, and miR-155 were significantly upregulated by ischemia in the control group compared to the sham group. In the case of miR-126, its expression did not increase during ischemia but showed a marked elevation after 30 and 60 min of reperfusion, suggesting a delayed activation consistent with an anti-inflammatory regulatory role. These findings indicate a disruption in the pro-/anti-inflammatory balance induced by I/R, with both harmful and protective responses being triggered over time.

When compared with our prior observations of proinflammatory cytokines such as tumor necrosis factor- α (TNF-α) and IL-1β in the context of lung transplantation [34], the overall miRNA profile again reflected a dominance of proinflammatory and proapoptotic signals, particularly due to miR-142-5p and miR-155. We hypothesize that the increased expression of miR-152 and miR-126 may represent a compensatory, physiological attempt to counterbalance inflammation. Interestingly, only miR-126 showed no significant change at the pre-reperfusion (PreRep) time point in the control group, suggesting that its peak expression occurs later than that of the proinflammatory miRNAs, possibly as part of a delayed feedback mechanism.

Lidocaine treatment clearly modulated both molecular levels of regulation. It partially inhibited the upregulation of the proinflammatory miRNAs miR-142-5p and miR-155, reducing their expression during both ischemia and reperfusion (at 30 and 60 min). In contrast, the increase in the anti-inflammatory miRNAs miR-152 and miR-126 was completely blocked in the lidocaine-treated group at PR60. These parallel effects on MAPK phosphorylation and miRNA expression indicate that lidocaine modulates inflammatory signaling through coordinated regulation of transcriptional and post-transcriptional mechanisms.

### 3.3. Integrated Mechanisms and Supporting Evidence from Previous Studies

The present findings support the hypothesis that miRNAs and multiple signaling pathways, including MAPKs (such as p-38-MAPK and ERK), PI3K, and AKT, interact closely in shaping the lung’s response to I/R injury. The coordinated timing of pro- and anti-inflammatory signals suggests a dynamic regulatory network, in which early proinflammatory triggers initiate a cascade that is subsequently modulated by compensatory anti-inflammatory mechanisms. Lidocaine appears to blunt both arms of this response by attenuating upstream inflammatory activation.

**Figure 7:**
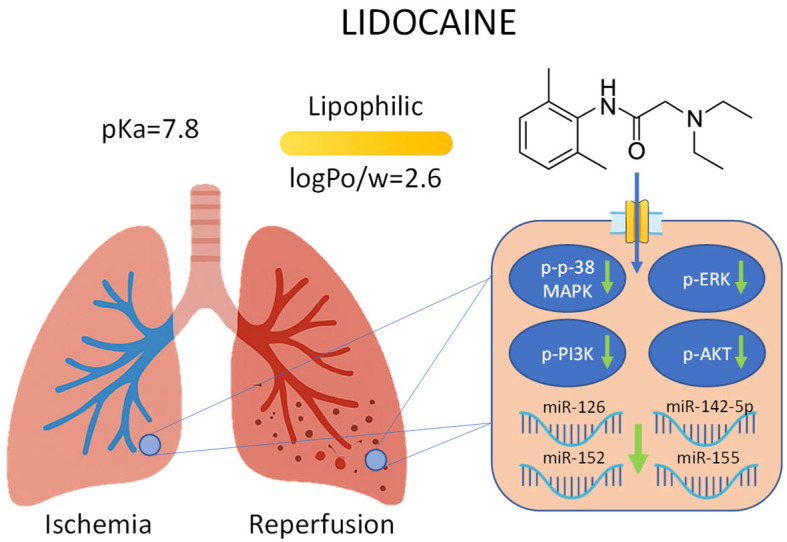
Schematic representation of the effects of intravenous lidocaine on kinase activation and miRNA expression during lung I/R injury. Lidocaine attenuates the phosphorylation of p-38 MAPK, ERK, PI3K, and AKT, while modulating the expression of miR-126, miR-142-5p, miR-152, and miR-155. These coordinated changes may reduce proinflammatory signaling while preserving cytoprotective pathways, contributing to improved graft protection. The green arrow indicates downregulation of the depicted elements.

These molecular observations are consistent with supporting evidence from previous studies performed using the same experimental model, which further characterized the mechanistic aspects of the I/R response and its modulation by intravenous lidocaine [24,35]. In those studies, hematoxylin–eosin staining revealed marked inflammatory infiltration and capillary congestion, predominantly involving monocyte–macrophage cells, findings that were reduced in animals treated with lidocaine. Immunohistochemical analysis showed increased expression of cluster of differentiation 68 (CD68) and monocyte chemoattractant protein-1 (MCP-1), together with decreased B-cell lymphoma 2 (Bcl-2) expression in untreated lungs, whereas lidocaine administration was associated with a lower degree of inflammatory changes and a higher number of Bcl-2-positive cells [35]. In parallel, biochemical analyses demonstrated elevated levels of inflammatory mediators, including TNF-α, IL-1β, and nuclear factor kappa-light-chain-enhancer of activated B cells (NF-κB), as well as activation of apoptotic markers such as caspases-3 and -9, Bcl-2-associated death promoter (Bad), and Bcl-2-associated X protein (Bax). Although the magnitude of these effects varied among parameters, lidocaine modulated the inflammatory and apoptotic responses induced by I/R [24]. Taken together, these previously published histological and biochemical results complement the current molecular data, suggesting that modulation of MAPK activation and miRNA expression may represent upstream regulatory events contributing to the downstream protective effects of lidocaine in this model (Figure 7).

### 3.4. Clinical Implications and Future Perspectives

The present results provide mechanistic insights with potential clinical relevance. Intravenous lidocaine is already used perioperatively for its analgesic, anti-inflammatory, and antiarrhythmic properties, and its safety profile is well established in humans. The observed modulation of MAPK activation and miRNA expression suggests that lidocaine may exert combined molecular benefits in I/R injury by attenuating proinflammatory signaling while preserving cytoprotective mechanisms. These effects could enhance graft viability and reduce early allograft dysfunction after lung transplantation. From a translational perspective, the experimental model employed in this study reproduces key aspects of the human surgical setting, thereby strengthening the clinical relevance of these findings. Future investigations should aim to validate these results in large-animal allotransplantation models and define optimal perioperative dosing regimens that balance systemic and local actions. Ultimately, early-phase clinical studies in lung transplantation or resection surgery are warranted to determine whether intravenous lidocaine can serve as a safe and effective adjunct to current strategies for limiting transplant-related lung injury.

## 4. Materials and Methods

### 4.1. Animal Model and Study Groups

The study was conducted at the Animal Facility and Experimental Surgery Unit of the Gregorio Marañón University General Hospital, Madrid, Spain. Eighteen male pigs (average weight: 36 ± 10 kg) were randomly assigned, using Excel for PC (Microsoft Corp., Seattle, WA, USA), to three experimental groups (*n* = 6 per group): lidocaine, control, and sham. Animals in the lidocaine and control groups underwent orthotopic left caudal lobe lung transplantation, whereas those in the sham group were subjected only to left thoracotomy without one-lung ventilation (OLV), pneumonectomy, or lobar reimplantation. In the sham group, both lungs were ventilated bilaterally throughout the procedure to avoid ischemia or reperfusion. This design allowed assessment of the physiological and molecular changes related solely to surgical handling and ventilation, thereby providing a baseline reference for comparison with the I/R and lidocaine-treated groups.

Each animal received an initial bolus of 1.5 mg/kg followed by a continuous infusion of 1.5 mg·kg^−1^·h^−1^, maintained until the end of the surgical procedure. The bolus and infusion in the lidocaine group contained lidocaine, whereas those in the control and sham groups contained 0.9% saline solution.

The lidocaine dosing regimen (1.5 mg/kg intravenous bolus followed by a continuous infusion of 1.5 mg·kg^−1^·h^−1^) was selected based on previously published pharmacokinetic and safety data from both experimental and clinical studies. In adult pigs, pharmacokinetic analyses have demonstrated predictable clearance and a hepatic extraction ratio of approximately 0.6 during intravenous lidocaine infusion, supporting the feasibility of continuous administration in this species [36]. Additional investigations in neonatal pigs have characterized lidocaine distribution and elimination kinetics, confirming its safety profile under controlled experimental conditions [37]. In human perioperative settings, comparable intravenous regimens have yielded mean plasma concentrations around 4 µg/mL (range 0.6–12.3 µg/mL), well within the established therapeutic range and below toxicity thresholds [38]. Although plasma lidocaine concentrations were not determined in the present study, the dosing protocol was designed to remain within pharmacologically safe and effective ranges documented in these prior investigations.

All syringes and infusions were prepared and administered in a blinded manner.

### 4.2. Surgical Procedure

Animals were fasted for 18 h before the procedure but had free access to water until 20 min prior to the intervention. Premedication was performed with intramuscular ketamine (10 mg/kg; Ketolar, Parke-Davis, Pfizer, Dublin, Ireland), followed by induction with propofol (4 mg/kg; Diprivan, AstraZeneca, Macclesfield, Cheshire, UK), fentanyl (3 µg/kg; Fentanest, Kern Pharmaceuticals, Houston, TX, USA), and atracurium (0.6 mg/kg; Tracrium, GlaxoSmithKline, Brentford, UK). Supplemental doses of fentanyl and atracurium were administered as required. Orotracheal intubation was performed, and volume-controlled mechanical ventilation was maintained throughout the procedure with a positive end-expiratory pressure of 5 cm H_2_O and peak inspiratory pressure below 30 cm H_2_O. Ventilation parameters included a tidal volume of approximately 8 mL/kg, a respiratory rate of 12–15 breaths/min, and an inspiratory-to-expiratory ratio of 1:2. The fraction of inspired oxygen (FiO_2_) was maintained at 1.0, and intraoperative crystalloid infusion was administered at a rate of 5–6 mL·kg^−1^·h^−1^. Anesthesia was maintained with continuous propofol infusion (8–10 mg·kg^−1^·h^−1^) in all animals, with lidocaine or saline administered according to group allocation described above.

**Figure 8:**
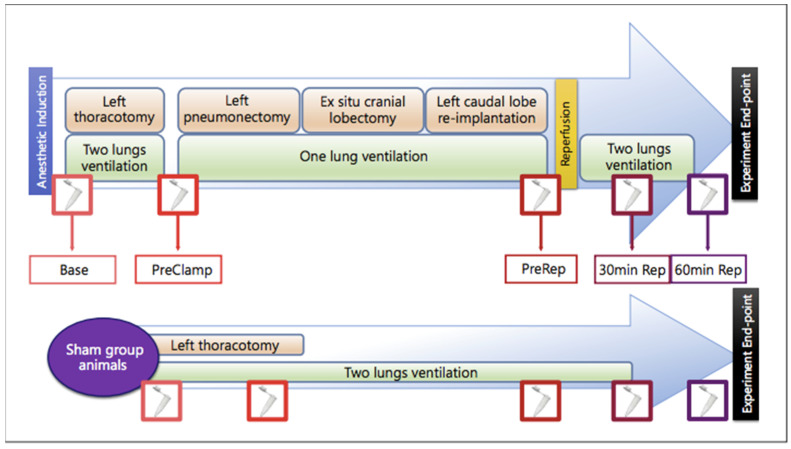
Schematic representation of the experimental surgical procedure and sampling time points. Base, baseline; PreClamp, before ischemia, i.e., before pulmonary artery clamping; PreRep, before reperfusion; 30 min Rep, 30 min after reperfusion; 60 min Rep, 60 min after reperfusion.

The surgical intervention consisted of a left thoracotomy through the fourth or fifth costal arch, followed by left pneumonectomy and ex situ cranial lobectomy. Animals were positioned in right lateral decubitus, and OLV ventilation was initiated under fiberoptic bronchoscopy, with tidal volume reduced to 6 mL/kg as part of pulmonary protection strategies. Systemic heparinization (300 IU/kg; Mayne Pharma, Madrid, Spain) was performed before pulmonary artery occlusion. The graft was perfused antegradely and retrogradely with University of Wisconsin solution (10–15 °C) while being ventilated manually with ambient air. The caudal lobe was then reimplanted by bronchial, arterial, and venous anastomoses, and graft reperfusion was initiated for 30 or 60 min, as illustrated in Figure 8. After the perfusion period, the animals were euthanized by deepening anesthesia followed by intravenous administration of potassium chloride to induce cardioplegia. The mean total procedure time was 289 min (range: 232–325 min), and the mean pulmonary ischemia time was 90 min (range: 84–97 min). Lung biopsies were collected at four time-points: prepneumonectomy (PreClamp, 5 min before pneumonectomy), prereperfusion (PreRep, 5 min before reperfusion), PR30 (30 min after reperfusion of the reimplanted lobe), and PR60 (60 min after reperfusion of the reimplanted lobe).

Detailed protocols for the anesthetic and surgical procedures used in this study have been described previously [27,35,39].

### 4.3. Hemodynamic and Arterial Blood Gas Analysis

For arterial blood gas analysis, PaO_2_, PaCO_2_, SaO_2_, and blood pH were measured from samples obtained from the femoral artery. Hemodynamic parameters, including MAP, HR and SVV were continuously monitored using the PiCCO system (Pulsion Medical Systems, Munich, Germany). Additional derived indices included CVP, CI, GEDVI, ELWI, and SVRI.

### 4.4. Western Blotting Analysis

Western blotting was performed to assess the protein expression levels of p-38 MAPK, ERK, PI3K, and AKT as previously described [40]. Four samples per time point and per group were analyzed. To assess the phosphorylated forms of p-38-MAPK, ERK, PI3K, and AKT (p-AKT^Thr308 and p-AKT^Ser473) tissue samples (50–60 mg) were homogenized in a lysis buffer and sonicated. To preserve protein phosphorylation, a phosphatase inhibitor was included during lysis. Protein concentrations were determined using the bicinchoninic acid (BCA) assay. Equal amounts of total protein (25 μg per sample) were denatured in loading buffer and separated on 10% SDS–polyacrylamide gels. Proteins were then transferred onto polyvinylidene difluoride (PVDF) membranes using a semi-dry transfer system.

Membranes were blocked at 37 °C for 1 h using PhosphoBLOCKER™ Blocking Reagent (Cell Biolabs, Inc., San Diego, CA, USA) to facilitate specific detection of phosphorylated proteins. After blocking, membranes were incubated with primary polyclonal antibodies at 4 °C for 12 h. Following incubation, membranes were washed three times with Tris-buffered saline with Tween-20 (TBS-T) under agitation. The TBS-T solution consisted of 10× TBS, Milli-Q water, and Tween-20 (PanReac Química, Barcelona, Spain).

Washed membranes were then incubated with horseradish peroxidase (HRP)-conjugated secondary antibodies (rabbit polyclonal) diluted 1:7000 in blocking buffer for 2 h at room temperature. After incubation, membranes were washed again in TBS-T under agitation. Protein bands were visualized using Clarity™ Western ECL Substrate (Bio-Rad Laboratories, Hercules, CA, USA; #1705061), following the manufacturer’s instructions. Chemiluminescence signals were detected using the ChemiDoc Imaging System (Bio-Rad Laboratories, Hercules, CA, USA), and bands were quantified with Image Lab 6.1 Software For Windows (Bio-Rad Laboratories, Hercules, CA, USA). Densitometry values were normalized to the total protein loaded in each lane. Four samples per time point and group were analyzed.

### 4.5. miRNA Extraction and Expression Analysis

miRNA was isolated from five independent lung samples using the mirVana™ miRNA Isolation Kit (Ambion, Life Technologies, Austin, TX, USA), following the small RNA enrichment procedure according to the manufacturer’s instructions, as previously described [27]. Briefly, RNA purity and concentration were assessed by spectrophotometry using a µL Biodrop (Isogen Life Science, De Meern, The Netherlands), and RNA integrity was confirmed by 40% acrylamide gel electrophoresis.

Reverse transcription of 350–1000 ng of miRNA was performed using the TaqMan^®^ MicroRNA Reverse Transcription Kit and Custom Reverse Transcription Pools, in combination with specific TaqMan^®^ MicroRNA Assays (Ambion, Life Technologies, Austin, TX, USA), following the manufacturer’s protocol. Quantitative PCR was carried out on an Applied Biosystems 7500 Fast Real-Time PCR System using TaqMan^®^ Universal Master Mix II (Applied Biosystems, Warrington, UK) and 1 µL of the corresponding 20× MicroRNA Assay.

To normalize cDNA input across samples, miR-103 expression was used as an endogenous control [41]. Relative expression changes were calculated using the 2^−ΔΔCT^ method [42].

### 4.6. Statistical Analysis

The Shapiro–Wilk test was used to test the normality of all measures, miRNA scores and kinase levels, at baseline (PreClamp time). Homogeneity of these measures for the lidocaine, control and sham groups at baseline was analyzed with one-way analysis of variance (ANOVA, SPSS v.27) F-tests. To investigate the effects of surgery I/R injury, and lidocaine, a repeated-measures ANOVA was performed for each variable. The between-subjects factor was group (sham, lidocaine and control), whereas the within-subjects repeated measures were scores in each time point (PreClamp, PreRep, PR30 and PR60). To correct for the multiple comparisons, Tukey post hoc analyses were used for all 3-comparisons between groups of animals. Differences were considered highly significant at *p* < 0.01 and very highly significant at *p* < 0.001. All data were analyzed using SPSS v.27 (SPSS Inc., Armonk, NY, USA).

## 5. Conclusions

Our study demonstrates that lung I/R injury led to the dysregulation of specific miRNAs and the activation of MAPK signaling pathways, contributing to a proinflammatory and proapoptotic environment. The temporal expression patterns observed suggest a complex interplay between pro- and anti-inflammatory responses, with miR-152 and miR-155 driving early inflammation and miR-126 and miR-142-5p potentially acting as delayed modulators. Lidocaine administration significantly attenuated these molecular alterations, reducing the expression of proinflammatory miRNAs and the phosphorylation of key MAPKs, particularly p-38 and ERK, as well as related kinases PI3K and AKT. These findings support the potential role of miRNAs and MAPKs as therapeutic targets in lung transplantation. However, the study had limitations, including the use of an experimental auto-transplant model and a focused panel of molecular markers. Moreover, as lidocaine was administered intravenously, its systemic effects—mediated through circulatory, neural, or immune pathways—cannot be completely separated from its local actions on the transplanted lung. However, this approach was deliberately chosen to reproduce the clinical conditions typically encountered during human lung transplantation, thereby enhancing the translational relevance of the model. In addition, miRNA expression was analyzed exclusively in lung tissue, as reliable isolation from porcine serum was not feasible under our experimental conditions. Consequently, the systemic circulating profile of these miRNAs could not be assessed, which limits the interpretation of their potential role as circulating biomarkers. Nonetheless, this limitation does not affect the validity of the observed tissue-specific molecular responses, which remain essential for understanding the local mechanisms of I/R injury. Overall, this work adds to the growing understanding of the molecular events underlying I/R injury and highlights lidocaine as a promising candidate for further investigation in strategies aimed at reducing transplant-related lung damage.

## Figures and Tables

**Table 1:** Relative Western blot expression levels of p-38 MAPK, ERK, PI3K, AKT, and their phosphorylated forms in the three experimental groups; glyceraldehyde-3-phosphate dehydrogenase (GAPDH) served as the loading control. PreClamp: before ischemia, i.e., before pulmonary artery clamping; PreRep: before reperfusion; PR30 and PR60: 30 and 60 min after reperfusion, respectively.

		Sham				Control				Lidocaine			
		Pre Clamp	Pre Rep	PR30	PR60	Pre Clamp	Pre Rep	PR30	PR60	Pre Clamp	Pre Rep	PR30	PR60
p-38 MAPK	43 kDa	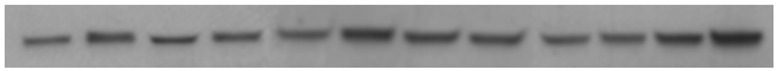											
p-p-38 MAPK	43 kDa	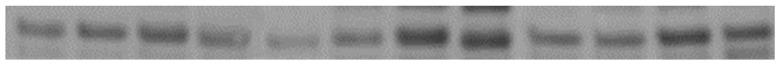											
ERK	42.4 kDa	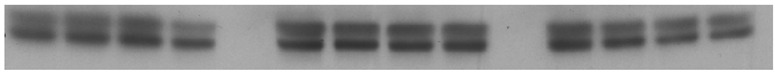											
p-ERK	42.4 kDa	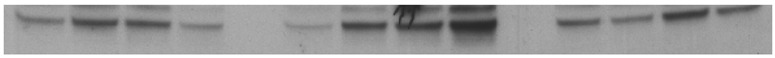											
PI3K	85 kDa	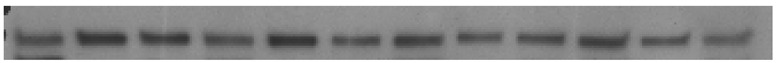											
p-PI3K	85 kDa	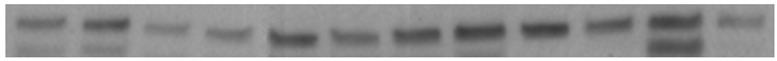											
AKT	60 kDa	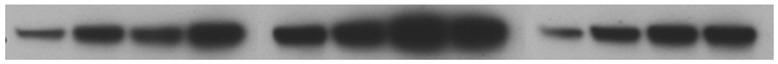											
p-AKT Thr	60 kDa	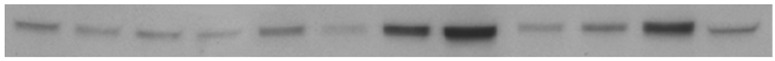											
p-AKT Ser	60 kDa	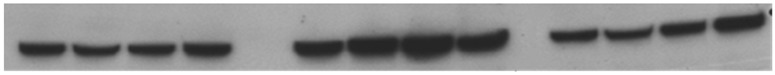											
GAPDH	37 kDa	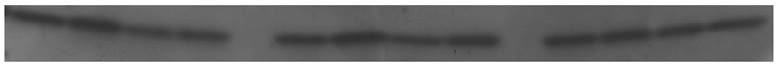											

## Data Availability

The data that support the findings of this study are available from the corresponding author upon reasonable request.

## Supplementary Materials

The following supporting information can be downloaded at: https://www.mdpi.com/article/10.3390/ijms262110385/s1.
