# Supplementary material for: Lidocaine Attenuates miRNA Dysregulation and Kinase Signaling Activation in a Porcine Model of Lung Ischemia/Reperfusion Injury
Source: Int J Mol Sci. 2025 Oct 25;26(21):10385. doi: 10.3390/ijms262110385 (PMC12607458; doi:10.3390/ijms262110385)
Supplement: Supplementary file 1 [file ijms-26-10385-s001.zip › Supplementary Table S1.pdf]

Hemodynamic variable	Group	PreClamp	PreRep	PR30	PR60
HR (bpm)	Sham	89.0 ± 24	87.6 ± 14	98.0 ± 18	97.2 ± 11
	CON	94.6 ± 14	90.4 ± 18	96.8 ± 8	96.8 ± 18
	LIDO	88.2± 13	104.3 ± 7.7	106.2 ± 10	96.6 ± 9
MAP (mmHg)	Sham	93 ± 11	94 ± 12	112 ± 14	104 ± 14
	CON	92 ± 17	100 ± 19	127 ± 16	106 ± 17
	LIDO	96 ± 14	98.6 ± 12	116 ± 9	100 ± 12
CVP (mmHg)	Sham	9.8 ± 3.6	10.3 ± 4.2	9.5 ± 5.8	6.9 ± 5.5
	CON	8.5 ± 5.7	7.6 ± 4.4	9.2 ± 4.8	8.8 ± 4.5
	LIDO	7.9 ± 6.2	10.2 ± 2.1	7.2 ± 6.2	7.8 ± 2.7
CI (L/min/m^2)	Sham	2.73 ± 0.9	2.89 ± 0.7	3.43 ± 1.2	3.05 ± 1.1
	CON	3.46 ± 0.8	3.12 ± 0.7	3.74 ± 1.4	2.74 ± 1.0
	LIDO	3.07 ± 0.9	3.13 ± 0.6	3.96 ± 1.5	2.8 ± 0.5
GEDVI (L/min/m^2)	Sham	497 ± 156	517 ± 156	589 ± 187	480 ± 126
	CON	546 ± 249	514 ± 258	614 ± 85	406 ± 117
	LIDO	585 ± 100	501 ± 104	545 ± 88	482 ± 89
ELWI (mL/kg)	Sham	8.5 ± 2.7	13.5 ± 5.7	14.3 ± 5.5	7.2 ± 2.4
	CON	17.0 ± 8.4	14.9 ± 8.5	18.8 ± 8.4	9.6 ± 3.5

	LIDO	16.8 ± 9.2	13.3 ± 5.2	14.2 ± 4.1	7.5 ± 2.5
SVV (%)	Sham	19.1± 5.5	12.1 ± 2.8	11.2 ± 5.5	18.0 ± 6.1
	CON	19.4 ± 6.1	15.3 ± 8.9	15.0 ± 3.7	17.6 ± 5.3
	LIDO	16.3 ± 4.4	12.3 ± 4.2	14.3 ± 1.2	16.8 ± 7.3
SVRI (dyn/s/cm^5/m^2)	Sham	2898 ± 929	3180 ± 533	3106 ± 383	3017 ± 604
	CON	1999 ± 596	2439 ± 675	2749 ± 505	2799 ± 864
	LIDO	2515 ± 597	3093 ± 619	2695 ± 677	2879 ± 838

Values were expressed as mean ± standard deviation. CON: control group; LIDO: lidocaine group; HR: heart rate; MAP: mean arterial pressure; CVP: central venous pressure; CI: cardiac index; GEDVI: global end-diastolic volume index; ELWI: extravascular lung water index; SVV: stroke volume variation; SVRI: systemic vascular resistance index.
