# Supplementary material for: Lidocaine Attenuates miRNA Dysregulation and Kinase Signaling Activation in a Porcine Model of Lung Ischemia/Reperfusion Injury
Source: Int J Mol Sci. 2025 Oct 25;26(21):10385. doi: 10.3390/ijms262110385 (PMC12607458; doi:10.3390/ijms262110385)
Supplement: Supplementary file 1 [file ijms-26-10385-s001.zip › Supplementary Table S2.pdf]

Gasometric variable	Group	PreClamp	PreRep	PR30	PR60
SaO_2 (%)	Sham	99.5± 1.2	100 ± 0.0	100 ± 0.0	100 ± 0.0
	CON	100 ± 0.0	99,9 ± 2.1	99.8± 0.9	99.9 ± 1.1
	LIDO	99.8 ± 0.1	99.8± 2.9	99.5 ± 1.3	99.8 ± 0.7
PaO_2 (mmHg)	Sham	280 ± 79	254 ± 50	255 ± 45	251 ± 54
	CON	237 ± 56	146 ± 25	236 ± 47	245 ± 58
	LIDO	263 ± 84	147 ± 28	235 ± 55	252 ± 46
$PaCO_2$ (mmHg)	Sham	43.1 ± 4.3	40.3 ± 3.4	44.3 ± 4.0	40.0 ± 6.7
	CON	42.4 ± 6.1	43.2 ± 6.8	46.4 ± 5.8	38.9± 8.7
	LIDO	41.5 ± 4.0	45.0 ± 4.9	46.5 ± 4.2	39.9 ± 3.1
pH	Sham	7.5 0.0	7.5 ± 0.0	7.5 ± 0.0	7.5 ± 0.1
	CON	7.5 ± 0.1	7.5 ± 0.1	7.5 ± 0.1	7.5 ± 0.1
	LIDO	7.5 ± 0.0	7.38 ± 0.2	7.41 ± 0.0	7.5 ± 0.0

Values were expressed as mean ± standard deviation. CON: control group; LIDO: lidocaine group; SaO_2: arterial oxygen saturation; PaO_2: partial pressure of oxygen; $PaCO_2$: partial pressure of carbon dioxide; pH: blood pH.
